# Distinct Toll-like Receptor 3 and 7 Expression in Peripheral Blood Mononuclear Cells From Patients with Chronic Hepatitis C Infection

**DOI:** 10.5812/hepatmon.16421

**Published:** 2014-04-07

**Authors:** Mahsa Motavaf, Fatemeh Noorbakhsh, Seyed Moayed Alavian, Zohreh Sharifi

**Affiliations:** 1 Department of Microbiology, Islamic Azad University of Varamin-Pishva, Varamin, IR Iran; 2 Department of Molecular Hepatology, Middle East Liver Disease Center (MELD), Tehran, IR Iran; 3 Blood Transfusion Research Center, High Institute for Research and Education in Transfusion Medicine, Tehran, IR Iran

**Keywords:** Hepatitis C, Chronic, Toll-Like Receptor 3, Toll-Like Receptor 7, Immunity, Innate, Gene Expression

## Abstract

**Background:**

Hepatitis C virus (HCV) is a major cause of chronic liver disease, with around 130 million infected people worldwide. HCV is recognized by Toll-like receptors (TLRs), which are key mediators of innate immune response. Up on activation of TLRs, anti-viral cytokines and pre-inflammatory are produced.

**Objectives:**

In this study, we compared the expression levels of two members of the TLR family (TLR3 and TLR7) that recognize viral RNA in peripheral blood mononuclear cell (PBMC) of patients with chronic HCV infection and healthy controls.

**Patients and Methods:**

In this case-control study, blood samples were collected from patients admitted to Blood Transfusion Research Center, Tehran, Iran. PBMC was isolated from blood of chronic HCV patients (n = 25) and age and sex-matched healthy controls (n = 25). RNA was extracted from PBMC and cDNA was synthesized from total RNA templates using reverse transcriptase. The relative level of expression was quantified by real-time PCR using Glyceraldehyde-3-phosphate dehydrogenase (GAPDH) as reference gene and the results were compared by Pfaffl method. Data were analyzed using non-parametric Wilcoxon test. P < 0.05 was considered significant.

**Results:**

In both groups, we had 13 males and 12 females with a mean age of 48.7 ± 16. TLR3 (6.23 ± 0.91 vs. 3.89 ± 0.85, P < 0.001) and TLR7 (1.48 ± 0.82 vs-1.33 ± 1.18, P < 0.001) expressions were significantly lower in patients with chronic HCV infection when compared with healthy controls.

**Conclusions:**

This study suggests that decrease in levels of TLR3 and TLR7 expression is a mechanism that may enable HCV to evade the host innate immune response.

## 1. Background

Available data indicate that up to 3% of the world’s population (170 million) has been infected with HCV. The prevalence of hepatitis C varies throughout different regions of the world. A study conducted in Iran showed that the prevalence of HCV infection in the general population is less than 0.5% (1). This virus is an enveloped, single-stranded RNA (ss-RNA) virus with positive polarity, which is a leading cause of liver diseases including hepatitis, liver cirrhosis and hepatocellular carcinoma. Several HCV-derived products, including HCV proteins and HCV RNA trigger host immune response. Both adaptive and innate responses are crucial in controlling and eliminating HCV infection. The host innate immune system detects this virus and responds to its stimuli, mainly through recognition of Toll-Like Receptors (TLRs). TLRs, are important components of innate immune response that recognize pathogen-associated molecular patterns (PAMPs) and trigger immune responses. Members of this family have emerged as key sensors of viruses, recognizing their components such as nucleic acids and proteins. Engagement of TLRs leads to a series of signaling events resulting in the production of IFN-α, IFN-β, IFN-γ, and other TLR-induced inflammatory cytokines. To date, 10 kinds of human TLRs have been identified, which are expressed mostly on cell types involved in immune function, such as monocytes, neutrophils and dendritic cells (2). TLRs are predominantly expressed on the cell surface (TLRs1, 2, 4, 5, 6 and 10). However, some TLRs such as TLR3, 7, 8 and 9 are localized in the endosomal compartments (3). Among the mammalian TLRs, three are responsible for recognition of RNA. TLRs 7 and 8 are sensors for viral, ss-RNA and TLR3 recognizes viral double-stranded RNA (dsRNA), which constitutes the genome of one class of viruses and is also generated during the life cycle of many other viruses. Upon binding to their ligands, these RNA recognizing receptors trigger the activation of Interferon-regulatory factor 3 (IRF-3) - a transcription factor playing a critical role in the induction of type I interferon. TLRs function through signaling processes that are coupled to the myeloid differentiation factor 88 (MyD88) adaptor protein (4), with the exception of TLR3, acting via Toll-interleukin-1 receptor-domain-containing adaptor inducing IFN-β (TRIF) adaptor protein (5).

Accumulating evidence indicates that there is a relationship between HCV and the TLR-mediated signaling pathways (6-8). As HCV is a ss-RNA virus, it is supposed to be susceptible to detection by TLR7 and 8. Furthermore, the replicative machinery of HCV generates ds-RNA replicative intermediate that is likely to be recognized by dsRNA-sensing receptor, TLR3. In over one-half of patients, acute infection evolves into a persistent carrier state, presumably due to the ability of HCV to limit the activation of the host immune mechanisms. Evasion of the immune response by HCV has been documented in several different studies (7). TLR-mediated proinflammatory cytokine responses are necessary for host defense against HCV. Current evidences suggest that some HCV products inhibit the TLR-dependent signaling pathway through interactions with the downstream adaptor molecules or alteration of TLRs expression levels (9, 10). This suggests that changing the TLR-mediated signals is one of the mechanisms of HCV-induced immune modulation.

## 2. Objectives

The aim of this work was to determine the relative levels of TLR3 and TLR7 expression in patients with and without chronic HCV infection. Indicating the possible effect of HCV infection on expression of these essential components of the innate immune response may provide a new set of molecular markers for the prognosis of the HCV infection and suggest additional anti-HCV targets.

## 3. Patients and Methods

### 3.1. Subjects

This case-control study was approved by Ethics Committee of Islamic Azad University (Code: 14330507902001), Varamin branch, Iran. Twenty-five subjects who had HCV chronic liver disease, admitted to the Blood Transfusion Organization Clinical Laboratory, Tehran, Iran, from March 2013 to September 2013 were selected in this study using convenient sampling method. The sample size was calculated as 25 subjects in each group in order to compare the two mean considering α = 5%, statistical power of 80%, and effect size 0.51 using paired sample size formula. They were seropositive for anti-HCV antibody as detected by enzyme-linked immunosorbent assay (ELISA) (Hepanostika HCV ultra, manufactured by Beijing United Biomedical Co. Ltd, China) and recombinant immunoblot assay (RIBA) (MP Biomedicals, So-lon, OH, United States). The presence of HCV RNA was conﬁrmed by reverse transcription-polymerase chain reaction (RT-PCR) kit (Roche Applied Sciences, RAS, Mannheim, Germany) according to manufacture recommendation. In order to avoid the possible effect of therapy or other infections on the expression of TLRs, patients who had received antiviral treatment and patients with HIV or HBV co-infections were excluded (n = 13) (11). Analyses of serum ALT levels in HCV patients were performed by one observer using the Hitachi 704 auto analyzer, (Tokyo, Japan) with Pars Azmoon reagents kit (Tehran, Iran). From the group of patients 12 had histologically or clinically confirmed cirrhosis. All of patients at the time of study had virmia and elevated levels of ALT. Twenty five age and sex-matched healthy subjects, seronegative for HCV and HBV, were enrolled as controls. All patients signed a written informed consent for the study.

### 3.2. Total RNA Extraction and cDNA Synthesis

Four-ml venous blood samples were collected into EDTA-containing tubes and PBMCs were separated by density gradient centrifugation. Then, total RNA was extracted using Trizol reagent (Invitrogen, Karlsruhe, Germany) and mRNA was purified with Oligotex kit (Qiagen, Courtaboeuf, France) according to the manufacturer’s instruction. The quantity and purity of extracted RNA was assessed by measuring absorbance at 260 nm and the ratio A260/A280 in a UV-spectrophotometer (NanoDrop Inc, Wilmington, DE USA). Only samples with an A260/A280 ratio up to 1.8 were considered valid for real-time PCR. Reverse transcription of 1 μg of RNA into cDNA was performed using Takara 1st strand cDNA synthesis kit (Takara, Bio Inc., Shiga, Japan), according to manufacturer’s recommendations.

### 3.3. Real-Time PCR

The expressions of target genes (TLR3 and TLR7) were quantified using the comparative threshold cycle (Ct) method where the amount of target mRNA was normalized to an internal control (GAPDH) and expressed relative to a calibrator sample (healthy group). The real-time PCR was performed using the LightCycler FastStart DNA SYBR-Green I kit (Roche Applied Sciences, RAS, Mannheim, Germany) according to the protocol provided in the kit. Specific primer sets (12, 13) optimized for the Light-Cycler, were developed and purchased from the Cinagene Company, Iran. At each amplification cycle, accumulation of PCR products was detected by monitoring the increase in fluorescence by dsDNA-binding SYBR Green. To control for specificity of the amplification products, after the final amplification cycle, dissociation/melting curve was generated in the range of 55˚C to 95˚C. To quantify the relative expression of each gene, we used comparative ∆∆Ct method (14) with the following formula: ∆Ct = Ct (target, TLR) - Ct (internal control, GAPDH), where Ct is the threshold cycle corresponding to the PCR cycle number at which fluorescence increases above a fixed threshold value. The ∆∆Ct method calculation involved finding the difference between mean value of the ∆Ct of HCV patients group and the mean value of the ∆Ct from calibrator group for each analyzed molecule (TLR3 and TLR7). Fold change in the expression of target mRNAs in HCV patients compared to healthy controls was calculated as 2-(∆∆Ct). As the ∆∆Ct method is only applicable when the amplification efficiencies of the target gene and the internal control gene are essentially equal (14), we prepared five serial ten-fold dilutions for TLR3, TLR7 and GAPDH genes. Then, a standard curve was established by plotting logarithm of copy numbers in dilutions against the ΔCt values (Ct target TLR - Ct internal control GAPDH). The difference in PCR efficiency was determined by calculating the slope of the line. All samples were tested in triplicate, and the average values were used for quantification.

### 3.4. Statistical Analysis

Data were analyzed with SPSS version 17 (SPSS Inc., Chicago, Ill., USA). Continuous variables are presented as mean ± standard deviation (SD) and median; inter quartile range (IQR). We used Kolmogorov–Smirnov test to find out if the recorded data are normally distributed. Paired sample t-test or Wilcoxon test were used for continuous variables. In this study, the probability value of 0.05 or less (P ≤ 0.05) was set to know the significance level.

## 4. Results

This study was conducted on 50 subjects (25 cases and 25 in control group). In each groups, we had 13 males and 12 females with a mean age of 48.7 ± 16. Evaluating the specificity of the amplification products by dissociation/melting curve did not show any amplification of unspecific products. The slopes of the resulting standard curves for TLR3 and GAPDH as well as TLR7 and GAPDH were < 0.1, indicating that amplification efficiencies are comparable. Analyzing real-time PCR data by the comparative ∆∆Ct method indicated that the expressions of TLR3 and TLR7 mRNA decreased more than five and seven-fold, respectively, compared with the healthy controls. The decrease of both TLR3 (P < 0.001) and TLR7 (P < 0.001) mRNA expressions were significantly different compared with the controls. The mRNA relative expression values for TLR3 and TLR7 are illustrated in Table 1. 

**Table 1.** Summary of Descriptive Difference Between ΔCt of HCV Patients and the ΔCt From Control Group for TLR3 and TLR7 ^a^

	Mean ± SD	Median	IQR	∆∆Ct	2-(∆∆Ct)	P value
**ΔCt_(TLR3)_**						< 0.001
HCV Patient	6.23 ± 0.91	6.42	1.39			
Control	3.89 ± 0.85	3.87	0.75	-2.34	2-2.34 ≈1/5	
**ΔCt**_** (TLR7)**_						< 0.001
HCV Patient	1.48 ± 0.82	1.59	1			
Control	-1.33 ± 1.18	-1.62	0.91	-2.8	2-2.8 ≈1/7	

^a^ Abbreviation: IQR, inter quartile range

## 5. Discussion

HCV infection is a world-wide problem with the majority of HCV-infected individuals become chronic carriers; however, the mechanism of progression to chronicity remains unresolved. HCV has been shown to evade host immune response, which appears to be crucial for viral persistence and development of chronic infection (12, 15, 16). One of the suggested mechanisms by which HCV is able to disrupt both innate and adaptive immune responses inhibit is modifying the expression of multiple defense genes in the host (6-8, 12). TLRs are a family of receptors that play key roles in innate immunity. Recent research indicates that the ligands for TLR3 and TLR7 can inhibit HCV replication in HCV-infected patients, suggesting that these TLRs may play a role in regulating HCV infection (17).

HCV products are able to stimulate TLR signaling; however, HCV is able to simultaneously evade immune response through targeting and impairing TLR signaling (7, 10). It is suggested that HCV interferes with TLR signaling pathway via interaction with their signaling intermediates (16, 18) or altering their expression levels (10, 12). The data presented in this study indicated that HCV downregulats the expression of TLR3 in PBMC of patients with HCV-infected chronic liver disease. TLR3 is a sensor for ds-RNA and plays a role in pathogenesis of HCV infection. Our data also showed that TLR7 mRNA is significantly downregulated in HCV patients compared to healthy controls. TLR7 recognizes the ss-RNA viruses including HCV. The ability of HCV to suppress the expression of TLRs, including TLR3 and TLR7, could underlie its success in establishing a chronic infection that ultimately ends in cirrhosis and hepatocellular carcinoma. Our results are in agreement with those of Atencia et al. study (9), in which the levels of TLR3 and TLR7 expression was reported to be significantly downregulated in patients with chronic HCV infection when compared with healthy controls. The results of a study conducted by Mohammed et al. (19) also supported our findings. A significant decrease of TLR7 expression in the presence of HCV infection was also identified in study of Chang et al. (10). The researchers indicated that HCV interferes with the TLR7 gene expression by induction of TLR7 mRNA instability. Sato et al. (20) also reported suppressed TLR3 expression in HepG2 cells transfected with the entire HCV gene. However, our results opposed with those published by Dolganiuc et al. (12), who reported upregulation of almost all TLRs (including TLR3 and TLR7) in monocytes and lymphocytes of patients with chronic HCV infection. This contradiction can be explained by a number of factors including difference in methodological approaches (cellular separation vs. total blood) and differences in clinical stage (none of the patients included in that work had cirrhosis at the time of study, but from the group of patients in our study, 12 had cirrhosis). Furthermore, different studies indicated that expression levels of TLR3 and TLR7 are strongly correlated with the expression level of IFN-α (9, 19). Reduced expression of TLRs (TLR3 and TLR7) on innate immune cells with subsequent decrease in IFN-α production suggests that new therapies that aim to increase the expression level of TLRs or their activity may help in treatment of HCV infection. Synthetic activators of certain TLRs induce type I IFNs, which are known to inhibit HCV replication. TLR stimulation can also exert an IFN-independent antiviral effect. Recently, TLRs agonists have been shown to have clinical efficacy against HCV (17). A synthetic TLR7 activator, SM360320, was indicated to reduce HCV mRNA and protein levels in isolated immortal epithelial-like hepatocellular tumorigenic (Huh-7) cells (21). In addition, TLR3 antagonists, such as dsRNA mimic Polyinosinic: polycytidylic acid (poly I:C), may be beneficial in treating HCV infection. These evidences suggest that activators of TLR3 and TLR7 can induce potent antiviral activity against HCV, which may results in compensation of their low concentration on immune cells. In conclusion, we showed that patients with chronic HCV infection express lower levels of TLR3 and TLR7. Our data may aid to understand the HCV-induced immune evasion mechanisms and suggest additional therapeutic targets. However, further researches covering patients with other stages of disease (like the acute phase) or patients who received anti-HCV therapies will be needed to confirm TLRs real relative expression.
